# RNA-Seq Highlights Molecular Events Associated With Impaired Pollen-Pistil Interactions Following Short-Term Heat Stress in *Brassica napus*

**DOI:** 10.3389/fpls.2020.622748

**Published:** 2021-01-07

**Authors:** Neeta Lohani, Mohan B. Singh, Prem L. Bhalla

**Affiliations:** Plant Molecular Biology and Biotechnology Laboratory, Faculty of Veterinary and Agricultural Sciences, The University of Melbourne, Parkville, VIC, Australia

**Keywords:** *Brassica napus*, heat stress, heatwaves, pollen-stigma interaction, plant reproduction, pollen, pistil, canola

## Abstract

The global climate change is leading to increased frequency of heatwaves with crops getting exposed to extreme temperature events. Such temperature spikes during the reproductive stage of plant development can harm crop fertility and productivity. Here we report the response of short-term heat stress events on the pollen and pistil tissues in a commercially grown cultivar of *Brassica napus*. Our data reveals that short-term temperature spikes not only affect pollen fitness but also impair the ability of the pistil to support pollen germination and pollen tube growth and that the heat stress sensitivity of pistil can have severe consequences for seed set and yield. Comparative transcriptome profiling of non-stressed and heat-stressed (40°C for 30 min) pollen and pistil (stigma + style) highlighted the underlying cellular mechanisms involved in heat stress response in these reproductive tissues. In pollen, cell wall organization and cellular transport-related genes possibly regulate pollen fitness under heat stress while the heat stress-induced repression of transcription factor encoding transcripts is a feature of the pistil response. Overall, high temperature altered the expression of genes involved in protein processing, regulation of transcription, pollen-pistil interactions, and misregulation of cellular organization, transport, and metabolism. Our results show that short episodes of high-temperature exposure in *B. napus* modulate key regulatory pathways disrupted reproductive processes, ultimately translating to yield loss. Further investigations on the genes and networks identified in the present study pave a way toward genetic improvement of the thermotolerance and reproductive performance of *B. napus* varieties.

## Introduction

*Brassica napus* L. (canola/rapeseed) is the third most important oilseed crop produced globally. Rising demand for canola/rapeseed oil for human consumption, industrial uses and bio-diesel production has led to a continuous expansion of its cultivating areas to comparatively drier regions, thereby increasing the exposure to unfavorable weather events (Jaime et al., 2018). The critical growth temperatures for canola range from 27 to 30°C (Kirkegaard et al., 2018; Lohani et al., 2020a). Like other crops, *Brassica* requires a specific number of heat units or growing degree days (GDD) for the onset of the flowering phase (Kjellström, 1993). *B. napus*, a temperate crop is especially susceptible to high-temperature events, particularly during reproductive stages (Angadi et al., 2000; Young et al., 2004; Aksouh-Harradj et al., 2006). Accumulation of excessive heat units during the anthesis results in decreased reproductive fitness, thus making it a crucial developmental period for yield determination in *B. napus* (Morrison and Stewart, 2002; Uppal et al., 2019). With frequently occurring heatwaves predicted in the future global climate change scenarios, it is becoming increasingly important to understand the severity of likely damage to reproductive fitness and yield in crop plants.

The intensity, timing, rate of temperature change, and duration of heat stress exposure controls the sensitivity of sexual reproduction to high temperature. High temperature disrupts the stages involved in sexual reproduction by initiating a series of physiological, molecular, cellular, and biochemical changes in crops (Lohani et al., 2020b). A survey of the heat stress regimes utilized for studying the pollen thermotolerance highlight a need for focusing on the impact of short-term heat stress events on *B. napus* and other crops (Mesihovic et al., 2016). Since even short-lived high-temperature spikes can have significant adverse effects on reproductive success, it is crucial to understand the underlying molecular basis short-term heat stress response during pollen-pistil interactions (Frank et al., 2009; Begcy et al., 2019). Exposure of plants to shorter pulses of heat stress during reproductive processes will not only help to understand the heat stress response during various reproductive stages but also the extent of vulnerability of the developing reproductive organs to heat stress regimes.

While high temperature is expected to affect male and female reproductive tissues concurrently, the available data mainly focusses on the effects of heat stress on pollen development and function. Nevertheless, there is a recent focus on the female reproductive organ sensitivity to high temperature in crops like sorghum, rice, maize, wheat, and tomato (Gonzalo et al., 2020; Jagadish, 2020; Lohani et al., 2020b). The outcomes of these studies reveal a considerable variation in heat stress response of pistil (stigma or ovary) across crops. The female reproductive organ or the pistil comprises of the stigma, style, and ovary. The stigma (receptive part) and style (transmitting tract) play crucial roles in triggering, promoting, and guiding the growth of pollen tubes toward female gametes within the ovule. The exposure to high temperatures can disrupt pollen-pistil interactions and fertilization, leading to decline in seed set and crop productivity. However, the molecular basis of heat stress response in the female reproductive tissues remains largely unexplored.

To address gaps in our understanding of effects of short-term heat stress events on reproduction, we investigated the effects of heat stress at 40°C for 4 h on pollen-pistil interactions by performing reciprocal crosses between non-stressed, and heat-stressed pollen and pistil. We further explored the changes in transcriptional patterns of mature pollen and pistil to unravel the molecular signatures in response to heat stress (40°C for 30 min). We have also attempted to integrate physiological effects with the corresponding transcriptome level changes in the gene expression patterns upon short term heat stress exposure. Our findings show that heat stress impairs both the pollen viability and the ability of the pistil to support pollen germination and tube growth. Our RNA-seq analysis highlights differential regulation of specific genes involved in pollen cell wall organization, water channel activity, ROS metabolism, fatty acid metabolism, phenylpropanoid biosynthesis, and genes involved in pollen-pistil interactions.

## Materials and Methods

### Plant Growth Conditions and Temperature Treatments

*Brassica napus* var. Garnet (AV Garnet) was chosen for this study is mid- to early-maturing variety is grown commercially across Australia. The plants were grown in a Thermoline growth cabinet (model TPG-2400-TH) at the Plant Growth Facility of The University of Melbourne, Australia. Control growth conditions were 23/18°C day/night; a photoperiod of 16/8 h light/dark, 200 μmolm^–2^s^–1^ light intensity and 60% humidity. The plants bearing secondary inflorescence (50 das) were exposed only once to 40°C for 4 h. The high-temperature treatment started 2 h after the beginning of the day. Each experiment had three biological replicates. Throughout each experiment, plants were spatially randomized weekly and were kept well-watered to minimize any effects associated with drought stress. Soluble nutrient fertilizer was applied directly to the soil once a week for each plant.

### Pollen Viability and Germination Assay

Pollen viability was evaluated by double staining with Fluorescein Diacetate (FDA) and Propidium Iodide (Regan and Moffatt, 1990). Anthers isolated from the buds of appropriate size (6–7 mm) were macerated gently to release pollen grains in the staining solution (20 μL: 10% sucrose, 1 μL: 2 mg/mL FDA and 2 μL: 1 mg/mL PI). The samples were kept in the staining solution in the dark at room temperature for 20 min and observed under a fluorescence microscope (Olympus BX60). The viability was presented as percentage (%) calculated by counting a minimum of 200 pollens from each sample.

For pollen germination, pollen grains were collected from flowers with freshly dehisced anthers after each treatment. The pollen grains were allowed to hydrate for 30 min after which they were brushed onto the surface of the freshly prepared solid pollen germination medium (100 g sucrose, 25 mg boric acid, 90 mg calcium chloride, 50 mg potassium nitrate and 100 mg of Tris dissolved in 500 mL of water; 1% agar was used for solidifying it) (Singh et al., 2008). The pollen grains were germinated for 4 h under high humidity (>70%) and light (200 μmolm^–2^s^–1^). After 4 h, the plates were observed under a microscope for scoring.

### Reciprocal Crosses

At least 20 immature flowers (those due to open the next day) were emasculated from two plants per replicate (total three replicates) for each reciprocal cross. Next day the appropriate numbers of plants were exposed to heat stress at 40°C for 4 h. Manual pollination was performed immediately after heat stress treatment. The following four combinations of reciprocal crosses of non-stressed (NS) and heat-stressed (HS), pistil (♀) and pollen (♂) were carried out: NS♀× NS♂, NS♀× HS♂, HS♀× NS♂ and HS♀× HS♂. After pollination, the plants were allowed to mature under control conditions until the completion of seed filling to observe seed set.

Pistils from each reciprocal cross were also collected 24 h after pollination and were fixed in Carnoy’s solution overnight. Then washed with distilled water, softened with 8 M NaOH solution overnight and stained with aniline blue solution (0.1% aniline blue in 0.1 M K_2_HPO_4_-KOH buffer, pH 11) for 3 h in complete darkness (Kho and Baer, 1968). The stained pistils were observed with a fluorescence microscope (Olympus BX60).

### Statistical Analysis

All experiments were performed in triplicates. Results were expressed as the mean ± SD of n replicates available per treatment. The data were analyzed using GraphPad Prism 8.2.1 (1992–2020 GraphPad Software, Inc.) software. Data analysis was done by performing Welch’s *t*-test to compare a time point at a given temperature with the control conditions, and the variance was considered unequal for the comparison. Significant differences among the treatment were considered at *p* < 0.05.

### Isolation of Mature Pollen Grains and Pistil (Stigma + Style) for RNA Seq

**FIGURE 1:**
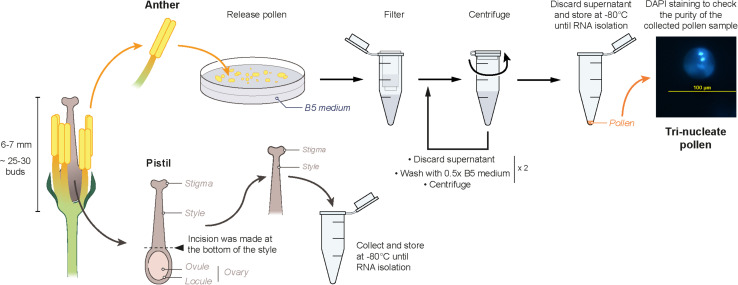
Schematic representation of the isolation method of mature pollen and pistil (stigma + style) for RNA extraction.

Mature flower buds (6–7 mm) were collected from non-stressed, and heat-stressed (40°C for 30 min) plants and transferred to a petri dish containing 0.5 × B5 medium and were kept on ice. Anthers carefully dissected out from the buds were lightly macerated in B5 medium. The crushed suspension was then filtered through a 44μm nylon mesh into 1.5 mL tubes. The filtrate was centrifuged at 150 g for 3 min at 4°C. The supernatant was discarded, the pellet was washed using 0.5 × B5 medium and centrifuged at 150 g for 3 min at 4°C. This step was repeated twice. After removing the supernatant, the pellet was immediately frozen in liquid nitrogen and stored at -80°C. An aliquot from each isolation was analyzed by nuclei staining with DAPI to check the purity of the sample for containing tri-nucleate pollen grains. While dissecting the buds for collecting anthers, the pistils were dissected out from the buds. An incision was made at the bottom of style using a scalpel for separating the upper part of the pistil from the ovary. The isolated stigma + style samples were collected in a 1.5 mL tube which was suspended in liquid nitrogen and then stored at -80°C (Figure 1). Twenty-five to thirty buds were collected from three plants as one biological replicate, and three independent biological replicates were prepared for each sample. To avoid confusion, the term “pistil” is used to refer to “stigma + style” samples throughout the article.

For RNA sequencing, heat-stressed pollen and pistil were collected from 50 days old plants immediately after exposure to heat stress at 40°C for 30 min. A 30 min heat stress treatment was selected to capture early heat stress-responsive changes in the transcriptome. Non-stressed pollen and pistil were collected from plants grown at control growth conditions.

### RNA Extraction, Library Preparation, and RNA-Sequencing

Total RNA was isolated from non-stressed and heat-stressed mature pollen as well as non-stressed and heat-stressed pistil samples (three biological replicates) using mirVana microRNA isolation kit (Thermo Fisher Scientific, Waltham, MA, United States) following the manufacturer’s instructions. The RNA samples were then shipped on dry ice to the BGI TECH SOLUTIONS (HONGKONG) CO., LIMITED where they underwent additional testing, according to their quality control pipeline for RNA Sequencing. The integrity of the RNA samples was analyzed using an Agilent Bioanalyzer 2100 before being confirmed as suitable to be run on a BGISEQ-500 platform for PE100 strand-specific mRNA sequencing with an expected output of 30 million raw reads per sample. After sequencing, the raw reads were filtered. Data filtering included removing adaptor sequences, contamination, and low-quality reads from raw reads. The read statistics of the RNA-Seq libraries are provided in Supplementary Table S1a.

### Transcriptome Analysis Pipeline

Quality checks for the raw fastq files were conducted using FastQC v0.11.8 (Andrews, 2010). Reference transcriptome file for *Brassica napus* was downloaded from Genoscope^1^. Transcript expression was quantified using Kallisto v0.44.0 (Bray et al., 2016). The transcript expression levels were converted to gene expression levels using tximport (Soneson et al., 2015) v1.6.0 (countsFromAbundance = “no”). The low-count genes were prefiltered by keeping only those genes that have at least 5 counts in total. The DESeq2 R package v1.28.1 was used to perform differential expression analysis. The tximport data was loaded into DESeq2 with DESeqDataSetFromTximport thus creating offset and correcting for changes to the average transcript length across samples (Love et al., 2019). Principal component analysis (PCA) was conducted to determine the relatedness of the biological replicates (Supplementary Figure S1A). Pairwise contrasts were performed between control and heat-stressed samples to identify differentially expressed genes (DEGs) in response to heat stress. To generate more accurate log_2_ foldchange estimates lfcShrink (type = “apeglm”) function was used. The thresholds for differential expression were set at fold change 1.5 and p-adjusted value cut off 0.01 (lfcthreshold = 0.585, altHypothesis = “greaterAbs,” alpha = 0.01, pAdjustMethod = “BH”) for the alternate hypothesis, BH: Benjamini-Hochberg (Love et al., 2019; Zhu et al., 2019).

### Functional Annotation

The DEGs were functionally annotated and enriched for GO terms using online tool PlantRegMap (Tian et al., 2020). This tool employs TopGO and incorporates GO annotations from TAIR 10, UniProt-GOA, InterProScan prediction and RBH-based experimental annotation transfer. The annotated GO terms were plotted using WEGO Tool, and the redundant enriched GO terms were removed and visualized by REViGO (Supek et al., 2011; Ye et al., 2018). Subsequently, pathway enrichment analysis of DEGs was carried out using the KOBAS 3.0 database (Xie et al., 2011). A GO term and a KEGG pathway were considered significantly enriched only when the corrected p-value for that pathway was <0.01 after applying Fisher’s exact test and false discovery rate (FDR; BH method) correction. Visualization of significantly enriched functional pathways was performed by ggplot2 R package (Wickham, 2016). Differentially expressed gene sets were used as input into SeqEnrich (Becker et al., 2017). SeqEnrich is a program adapted for *B. napus*, and it contains the most current information on *B. napus* TFs, promoter motifs, and gene ontology (GO) available, and uses these data to produce predictive regulatory networks. Networks produced with SeqEnrich were visualized in Cytoscape (Smoot et al., 2011). Based on the gene descriptors, the logos of TF binding motifs were downloaded from JASPAR^2020^ database^2^ (Fornes et al., 2020). Homologous *B. napus* genes, in comparison to the Arabidopsis proteome, were identified using the BlastP program with an E-value ≤ 1e-05. R packed Complexheatmap package was used to generate heat maps of gene expression (Gu, 2015).

## Results and Discussion

### Short-Term Heat Stress Events Negatively Regulate Reproductive Fitness in *B. napus*

**FIGURE 2:**
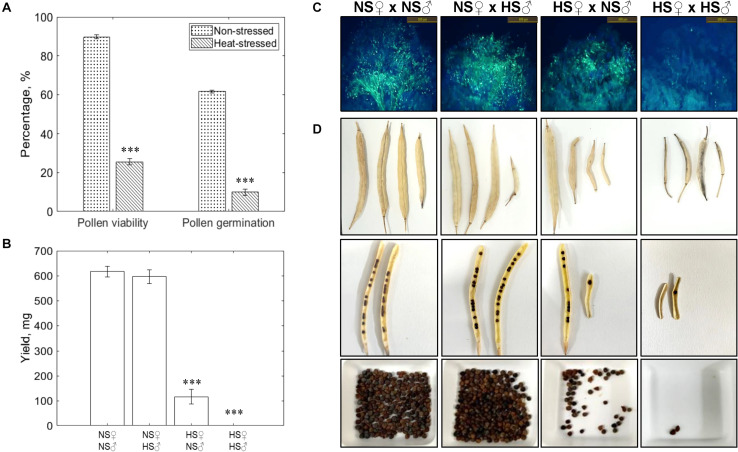
Impact of short-term heat stress on pollen-pistil interactions. **(A)** Pollen viability (%) and pollen germination (%) of non-stressed, and heat-stressed (40°C for 4 h) pollen. **(B)** Seed yield, mg produced from the fertilization of pistils pollinated during reciprocal crosses. **(C)** Aniline blue staining of reciprocal crosses (NS♀× NS♂, NS♀× HS♂, HS♀× NS♂ and HS♀× HS♂) to observe pollen-pistil interactions. **(D)** Siliques, seed filling of siliques, total seeds produced by the reciprocal crosses. Data are represented as the mean of three replicates ± SEM (standard error of the mean). The asterisk (*) represents a significant difference between heat stress treatment and non-stressed control (^∗∗∗^*P* ≤ 0.001).

To study the impact of short-term heat stress (4 h) on mature pollen, we exposed the *Brassica napus* plants bearing secondary inflorescences to 40°C for 4 h during the day. The pollen grains of non-stressed plants exhibited ∼90% pollen viability and ∼62% *in vitro* pollen germination (Figure 2A). Exposure to a short high temperature spike drastically reduced the pollen viability to ∼25% and pollen germination to ∼10%. Reciprocal crosses between non-stressed (NS), and heat-stressed (HS; 40°C for 4 h) pollen (♂) and pistil (♀): NS♀× NS♂, NS♀× HS♂, HS♀× NS♂ and HS♀× HS♂ revealed drastic reduction in pollen adhesion and germination on the stigma surface when heat-stressed (HS) pistils were pollinated with heat-stressed (HS) pollen, led to the failure of fertilization and seed set (Figures 2B–D). The highest seed set was recorded in, non-stressed (NS) pistil × non-stressed (NS) pollen cross. On the other hand, no significant reduction in seed set was noticed in NS pistil × HS pollen cross. The higher rate of seed set in NS pistil × HS pollen indicate that even ∼25% viable pollen grains (heat-stressed at 40°C for 4 h) were sufficient to fertilize almost all of the ovules. However, the seed set was reduced by ∼70% in HS pistil × NS pollen cross (Figure 2B). Relatively few non-stressed pollen grains, were successful in attaching to the heat-stressed stigma surface and then further germinating (Figure 2C) suggesting that non-stressed pollen is exposed to a unfavorable environment on the heat-stressed stigma surface leading to impaired interaction and reduced fertility. Thus, heat-induced changes in the pistil likely exert considerable influence over pollen performance and thereby the overall seed yield. Since plants produce abundant pollen, only a small percentage of viable pollen is sufficient for successful fertilization (Larden and Triboi-Blondel, 1994). On the other hand, due to relatively small number of ovules, heat stress sensitivity of pistil can have far severe implications for seed set and yield.

### Differential Transcriptional Response to Heat Stress in the Upper Part of the Pistil and Mature Pollen

**FIGURE 3:**
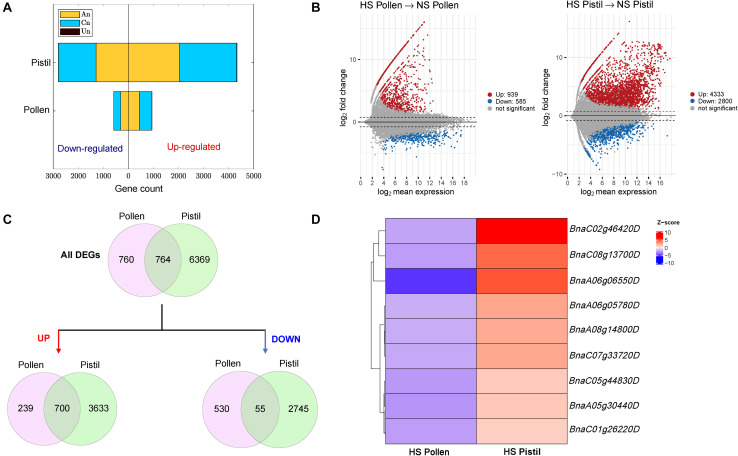
Differential gene expression analysis. **(A)** Number of up and down-regulated gene in heat-stressed pollen and pistil and their distribution on the A_n_ and C_n_ subgenome (Un represents genes located on uncertain chromosomes. **(B)** MA Plot of the differentially expressed genes in heat-stressed pollen and pistil; red dots represent upregulated genes, blue dot represent downregulated genes; and gray dots represent non-significant genes. HS, heat stressed; NS, non-stressed. **(C)** Venn diagrams showing all shared DEGs, only upregulated shared DEGs and only down-regulated shared DEGs between heat-stressed pollen and pistil. **(D)** Details and expression levels of the shared DEGs with upregulated expression in heat-stressed pistil and downregulated expression in heat-stressed pollen.

To explore the gene networks underlying heat stress responses in pollen and pistil, we performed heat stress treatments at 40°C for 30 min and employed a high-throughput strand-specific RNA-seq approach. The clean reads were uniquely aligned (Supplementary Figure S1B) to the reference assembly with a rate of 72.1% (median). The biological replicates for each sample clustered together and four clusters representing non-stressed pollen, heat-stressed pollen, non-stressed pistil, and heat-stressed pistil were identified in the PCA plot (Supplementary Figure S1A). The PCA plot highlighted the similarity of gene expression in the biological replicates for each sample. The relative transcript abundance in terms of TPM (transcripts per million) across all four samples (three biological replicates) are provided in Supplementary Table S1b (non-stressed and heat-stressed pollen) and Supplementary Table S1c (non-stressed and heat-stressed pistil). The threshold for differential expression was set at fold change 1.5 (log_2_foldchange = 0.585) and the adjusted p-value cut off 0.01 for an alternate hypothesis. Heat stress treatment of pollen led to differential expression of 1,524 genes comprising 939 up- and 585 down-regulated genes (Supplementary Table S1d). On the other hand, heat stress treatment of pistil revealed a total of 7,133 differentially expressed genes, including 4,333 up- and 2,800 down-regulated genes as compared with control treatment (Supplementary Table S1e). *B. napus* is an allotetraploid formed as a result of spontaneous hybridization between its two diploid progenitors: *B. rapa* (A genome, AA, 2n = 2x = 20) and *B. oleracea* (C genome, CC, 2n = 2x = 18). The genome composition of *B. napu*s is AACC (2n = 4x = 38). Subtle differences in the distribution of DEGs on sub-genome A_n_ and sub-genome C_n_ in both heat-stressed pollen and pistil was observed (Figure 3A). Recent reports are suggesting a sub-genome bias in *B. napus* favoring the expression of A_n_ sub-genome (Khan et al., 2020; Li et al., 2020). However, a further detailed investigation is required to improve the understanding of observed sub-genome expression dominance in *B. napus*.

Interestingly, heat stress-responsive up-regulated transcripts also demonstrated a greater relative change than down-regulated genes (Figure 3B). Comparison of the DEGs in response to heat stress in pollen and pistil, highlighted 764 shared DEGs (Figure 3C), out of which 755 DEGs showed similar regulation patterns (700 up- and 55 down-regulated in both). In contrast, nine shared DEGs were up-regulated in heat-stressed pistil and down-regulated in heat-stressed pollen (Figure 3D and Supplementary Table S2a).

Heat stress results in structural changes in proteins, accumulation of misfolded proteins, and various downstream ramifications for cellular homeostasis and development. To counter these effects, heat stress response through chaperone/heat shock protein (HSP)–heat shock factor (HSF) complexes is activated (Bokszczanin et al., 2013; Berz et al., 2019). Transcriptional reprogramming at higher temperatures is regulated by heat stress transcription factors (HSFs) leading to activation of a heat stress response. Based on the reports identifying the members of HSF gene family in *B. napus* (Zhu et al., 2017; Lohani et al., 2019), we detected the up-regulation of several members of the *B. napus* HSF gene family in heat-stressed pollen and pistil, except for HSFC1 (*BnaC07g07130D*) which was downregulated in pistil upon heat stress exposure. *B. napus* HSFA2 (*BnaC03g26940D, BnaA03g22890D*), HSFA7 (HSF7a: *BnaA03g41550D, BnaC07g32600D, BnaA03g41540D*, HSF7b: *BnaA09g40360D*) and HSFB2a (*BnaC03g52080D*) were the key HSFs upregulated in both pollen and pistil with >100-fold change in expression levels. Further, several differentially regulated HSPs and other chaperone encoding genes (ClpB1, DnaJ-domain chaperone, BAG6, ClpB3, ClpB4) were detected in heat-stressed pollen and pistil (Supplementary Tables S2b). In pollen, all the identified HSPs and chaperones were upregulated by heat stress. Similarly, in the heat-stressed pistil, majority of HSPs and chaperones were upregulated except *BnaC06g39440D* (Chaperone DnaJ-domain superfamily protein), *BnaA06g28870D* (Prefoldin chaperone subunit family protein), *BnaA02g11050D*, *BnaA03g12820D* (BAG family molecular chaperone regulator 1) and *BnaC05g29580D* (HSP). Small heat shock proteins (15.7, 17.6, 17.8, 18.5, 21, 22, 23.6, and 26.5 kDa HSPs) along with Hsp70b were among the upregulated HSPs in both heat-stressed pollen and pistil. Thus, the accumulation of HSFs, HSPs (sHSP, HSP70, HSP90, and HSP100) and chaperones in response to heat stress, illustrate the ability of pollen and pistil to activate “classical” heat stress-responsive mechanisms (Bokszczanin et al., 2013; Fragkostefanakis et al., 2015; Lohani et al., 2020b).

#### Functional Annotation

**FIGURE 4:**
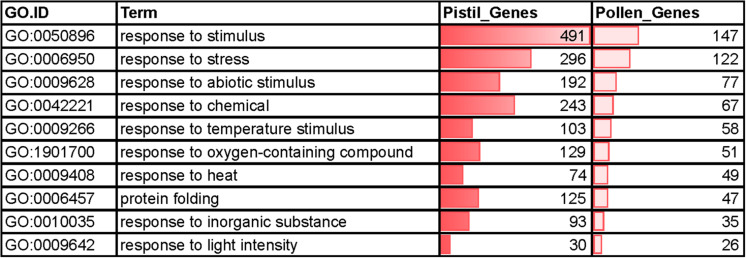
Top significantly enriched common GO terms associated with upregulated genes in heat-stressed pollen and pistil (*q* < 0.001).

Gene ontology (GO) analysis of the DEGs was performed to unravel their role in heat stress response (Supplementary Figure S2). Notably, 1,041 (642 up- and 499 down) and 5,192 (3,091 up- and 2,101 down) differentially regulated genes were assigned GO annotations in pollen and pistil, respectively. Analysis of enriched GO terms (*q* < 0.01) revealed that the upregulated genes in heat-stressed pollen and pistil were associated with abiotic stress associated GO terms like “response to stress,” “response to heat,” “response to stimulus,” and “protein folding” (Figure 4).

**FIGURE 5:**
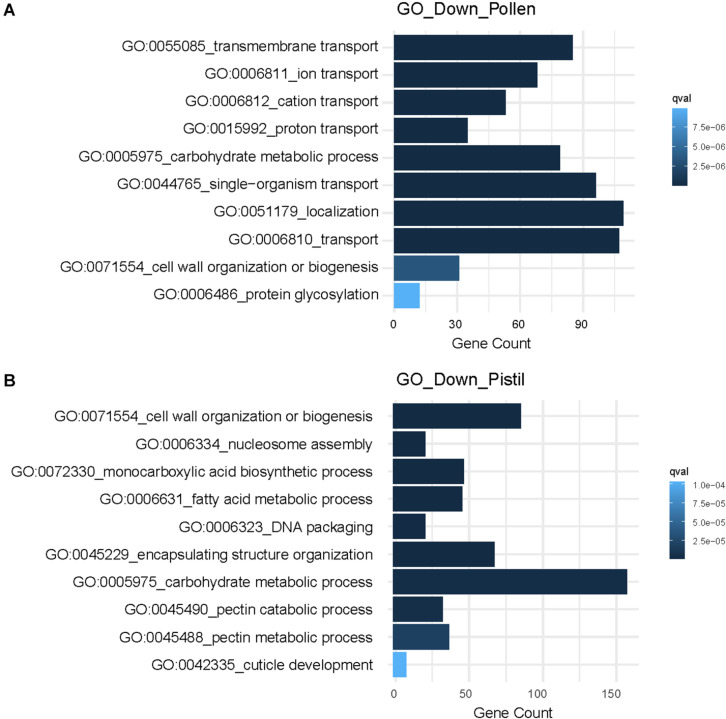
GO enrichment analysis of downregulated DEGs showing top 10 significantly. **(A)** Enriched GO terms associated with downregulated genes in heat-stressed pollen. **(B)** Enriched GO terms associated with downregulated genes in the heat-stressed pistil.

Further, the most significantly enriched downregulated GO terms in pollen were associated with “ion transmembrane transport,” “carbohydrate metabolic process,” “localization,” and “glycosylation” (Figure 5A). In heat-stressed pistil, the downregulated GO terms were related to “fatty acid metabolism,” “cell wall organization,” “response to abiotic stimulus,” “cuticle development,” and “lipid transport” (Figure 5B). Heat responsive down-regulated genes involved in cellular transport, carbon and nitrogen metabolism, cell organization and growth, and metabolic processes determine long-term adverse effects and are reported to differ significantly depending on the developmental stage or tissue exposed to heat stress (Janni et al., 2020).

**FIGURE 6:**
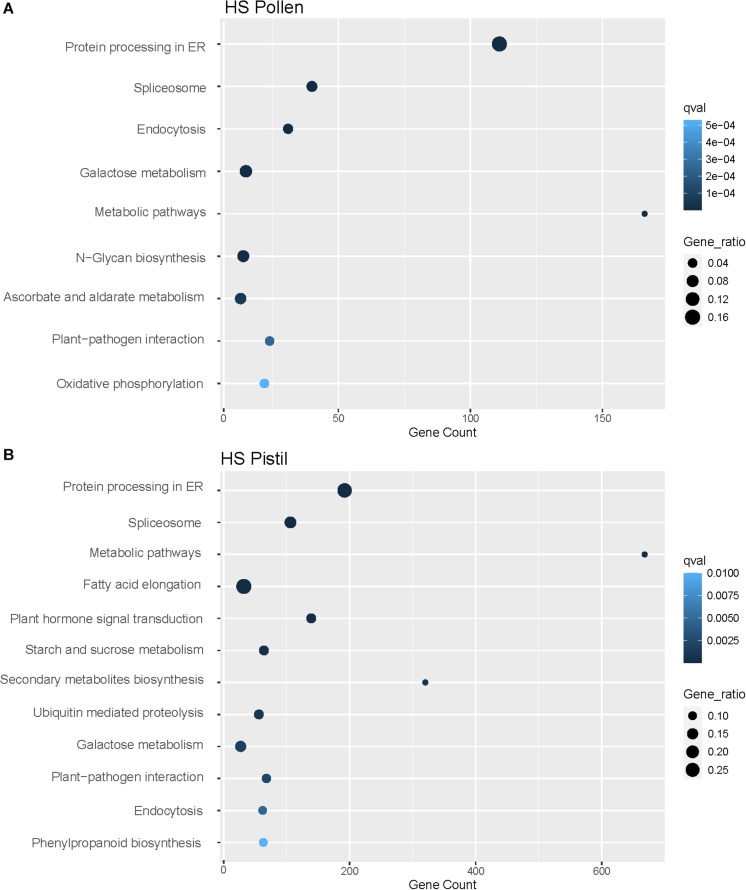
**(A)** Enriched KEGG pathways (*p* < 0.01) associated with the DEGs in heat-stressed (HS) pollen. **(B)** Enriched KEGG pathways (*p* < 0.01) associated with the DEGs in heat-stressed (HS) pistil.

Further, the analysis of enriched KEGG pathways in both pollen and pistil revealed differential upregulation of diverse pathways including protein processing in the endoplasmic reticulum (111 genes in pollen, 192 genes in pistil), spliceosome (40 genes in pollen, 106 genes in pistil), metabolic pathways (166 genes in pollen, 668 genes in pistil), and plant-pathogen interaction (24 genes in pollen, 68 genes in pistil) among other pathways (Figure 6). Many of these enriched functional gene categories and pathways have been linked with heat stress response or thermotolerance across different tissues in crops such as rice, wheat, maize, and tomatoes, which indicates a conserved mechanism of heat stress response in plants (Janni et al., 2020). We further explored the role of the functionally annotated DEGs associated with different metabolic and cellular processes to explore their role in regulating reproductive fitness and pollen-pistil interactions.

### Heat Stress Altered Cellular and Metabolic Processes Impair Pollen-Pistil Interactions

**FIGURE 7:**
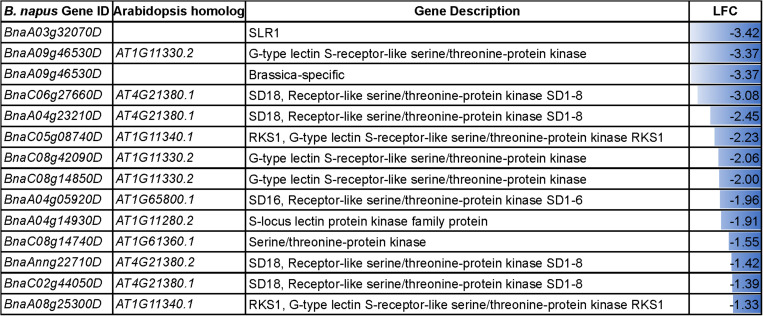
Details of the DEGs associated with the GO terms “recognition of pollen” and “pollen-pistil interactions” in the heat-stressed pistil. LFC, log_2_foldchange.

The acceptance of compatible pollen by the receptive pistil and the subsequent steps leading to successful fertilization involves interactive processes. The result of reciprocal crosses (NS♀× NS♂, NS♀× HS♂, HS♀× NS♂ and HS♀× HS♂) in our study highlighted that heat stress has a negative influence on the pollen-pistil interaction resulting in reduced seed set. In our datasets, the DEGs involved in the GO category “recognition of pollen” and “pollen-pistil interactions” were downregulated in the heat-stressed pistil (Figure 7). These heat-responsive genes might interfere with the attachment of pollen on to the stigma surface. For instance, the S-locus related gene, *SLR1* (*BnaA03g32070D*) belonging to the downregulated category, is reported to play a role in pollen adhesion (Luu et al., 1997, 1999). Further, genes involved in “plant-pathogen interaction (bna04626)” and “phenylpropanoid biosynthesis (bna00940)” were also differentially regulated in heat-stressed pollen and pistil. The differential regulation of phenylpropanoid biosynthesis pathway leads to accumulation of the intermediates (Supplementary Figure S3) which has been suggested to result in incompatible pollen-pistil interactions (Elleman and Dickinson, 1999).

Following pollen adhesion, the success of pollination and pollen tube growth depends on pollen hydration. In pollen very-long-chain fatty acids (VLCFA) are essential components of the pollen coat, facilitating interactions between pollen and stigma (Heizmann et al., 2000) and genes belonging to 3-ketoacyl-CoA synthase family are involved in their biosynthesis (Han et al., 2001). In heat-stressed pollen, five and in the heat-stressed pistil, twenty-nine genes homologous to 3-ketoacyl-CoA synthase genes were downregulated, suggesting downregulation of VLCFA synthesis due to heat stress (Supplementary Tables S3a, S4a).

Since lipid biosynthesis is essential for all aspects of pollen development, germination and pollen tube penetration of pistil tissues (Evans et al., 1992; Piffanelli et al., 1997), the observed alteration of genes expression involved in the fatty acid synthesis and lipid transport in heat-stressed pollen and pistil has apparent implications for pollen fitness and pollen-pistil interactions.

**FIGURE 8:**
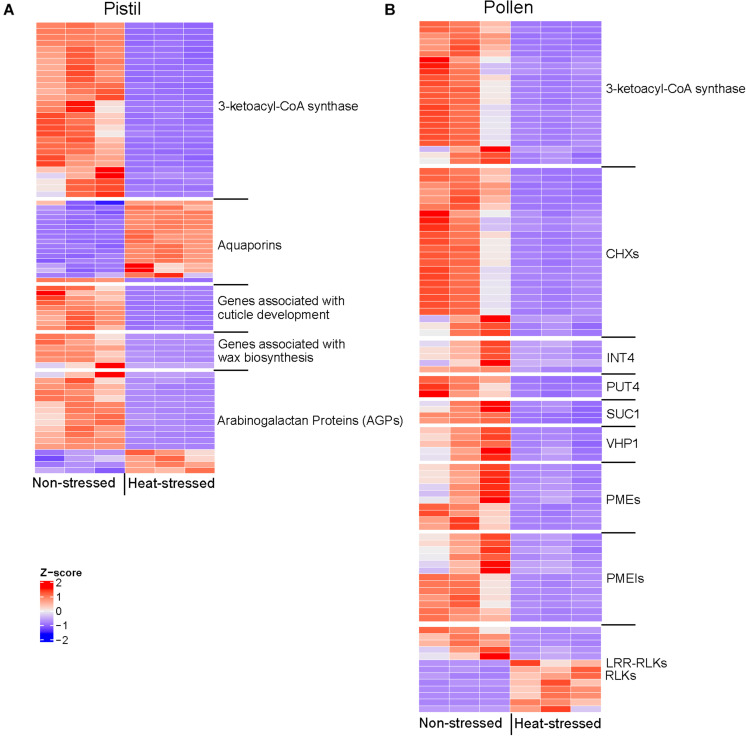
**(A)** Expression of selected genes differentially regulated in heat-stressed pistil possibly involved in pollen-pistil interactions. **(B)** Expression of selected genes differentially regulated in heat-stressed pistil possibly involved in pollen-pistil interactions. See the summary in Supplementary Tables S3a–f, S4a–f for log_2_foldchange.

Additionally, in heat-stressed pollen the GO categories “water channel activity” and “water transmembrane transport” were downregulated; especially genes homologous to Aquaporin TIP1-3 (*BnaA09g51590D*, *BnaCnng01570D*), indicating possible interference of the pollen hydration process (Supplementary Table S3b). In Arabidopsis TIP1-3 (*At4g01470*) aquaporin, is selectively expressed in pollen (Soto et al., 2008). A possible role of aquaporin-like genes in pollen hydration has also been previously suggested in *Brassica* (Ikeda et al., 1997). Here, we also observed the upregulation of genes homologous to Aquaporin PIP2-7, TIP1-1, and TIP2-1 in the heat-stressed pistil (Figure 8A and Supplementary Table S4b). PIP aquaporin genes are expressed in the stigma of *Brassica* (Dixit et al., 2001). In tobacco, PIP genes are suggested to be involved in the movement of water between pollen and stigma (Bots et al., 2005). However, the role of TIP1-1 and TIP2-1 in pistil remains elusive (Pérez Di Giorgio et al., 2016).

The stigma in *B. napus* is dry, and the stigmatic papillae are protected from dehydration and environmental conditions by waxy cuticle of the highly modified epidermal cells. Mutations that disrupt the formation of cuticles can have a profound impact on the ability of stigma to support pollen tube growth (Ingram and Nawrath, 2017). In heat-stressed pistil, genes involved in cuticle formation were also downregulated (Figures 5B, 8A and Supplementary Table S4c). Our data suggest that the downregulation of the genes associated with the biosynthesis of cuticle constituents in response to heat stress in pistil can interfere with pollen hydration and further germination.

Following hydration, pollen germinates on the stigma surface penetrating the stigma cells and then growing within the style’s transmitting tract. During growth toward ovary, the pollen tubes interact intimately with the extracellular matrix (ECM) components of the transmitting tract (Cheung et al., 1995). Hydroxyproline-rich glycoproteins (HRGPs), such as arabinogalactan proteins, AGPs (Cheung and Wu, 1999) in the extracellular matrix (ECM) have been suggested to regulate pollen tube growth and pollen tube guidance in the style tissue and regulate other functions, such as maintaining cellular integrity and cell-cell communications (Palanivelu and Preuss, 2000; Losada et al., 2017). Accordingly, we identified several differentially regulated AGPs like AGP4, AGP12, AGP15 and AGP18 in the heat-stressed pistil (Figure 8A and Supplementary Table S4d). Further research is required to explore the impact of heat stress on the HRGP mediation of pollen-pistil interactions in styler tissues.

### Pollen Cell Wall Organization and Cellular Transport-Related Genes Possibly Regulate Pollen Fitness Under Heat Stress

A significant percentage of downregulated genes in heat-stressed pollen was associated with the cell membrane components in comparison to heat-stressed stigma/style tissues. Key gene families involved in cellular transport such as cation/H^+^ antiporters, cation/H^+^ exchangers (CHXs), inositol transporters (INT4), sucrose transporter, SUC1, Na^+^/Ca^2+^ exchanger, proline transporter (PUT4), plasma membrane-associated ATPase, vacuolar H^+^ pyrophosphatases (VHP1), metal transporters and K^+^ uptake permease were downregulated among other transporters in heat-stressed pollen (Figure 8B and Supplementary Table S3c). While the possible role of CHX transporters in pollen function is not fully characterized, the Arabidopsis, *cxh17/18/19* mutants display a disordered architecture of the pollen wall, reduced male fertility and seed set (Chanroj et al., 2013). Similarly, *chx21/23* double mutant display male-sterility as the pollen tube is incapable of reaching the ovules (Lu et al., 2011). There is a recent report of the downregulation of these transporters in *in vitro* germinated Arabidopsis pollen exposed to heat stress (Poidevin et al., 2020). Sucrose transport gene *AtSUC1* shows high expression in pollen. The Arabidopsis, At*suc1* mutant pollen is defective *in vivo* with slower rates of pollen germination *in vitro* (Sivitz et al., 2008). Recently, the role of plasma membrane-associated ATPases in maintaining plant fertility was reported as these transporters control the ion balance by controlling downstream pH-dependent mechanisms essential for pollen tube growth (Hoffmann et al., 2020). Thus, based on our analysis and previous reports, we suggest that the downregulation of key genes involved in transmembrane transport is one of the key reasons for the loss of pollen function under heat stress conditions (Frank et al., 2009; Giorno et al., 2010; Fragkostefanakis et al., 2016; Keller et al., 2018; Gonzalo et al., 2020).

We also investigated heat-responsive pollen DEGs associated with cell wall organization to explore their role in pollen fitness. In our analysis, *BnaA02g34360D* encoding gene homologous to cellulose synthase CESA-6 is downregulated in heat-stressed pollen. In Arabidopsis, subunits of cellulose synthase complex CESA1-, CESA3-, and CESA6-related genes are involved in cellulose synthesize at the plasma membrane. Exclusion of any of these components leads to gametophytic lethality, indicating that primary-wall cellulose synthesis is crucial for pollen development (Persson et al., 2007). Thus, the downregulation of CESA-6 in heat-stressed pollen points toward inhibition of cellulose biosynthesis as one of the heat-induced pollen defects.

*B. napus* genes encoding probable pectin methyl esterases (PMEs) and PME inhibitors (PMEIs) were differentially regulated in heat-stressed pollen (Figure 8B and Supplementary Table S3d). PME and PMEI are known to regulate the stability of certain pollen tube wall domains during its elongation (Guan et al., 2013). Furthermore, in pollen crucial role of serine-threonine kinases and leucine-rich repeat receptor-like kinases (LRR-RLKs) in regulating pollen germination, pollen tube growth, and/or pollination has been suggested (Kim et al., 2004) and several members of the RLK gene family were also differentially regulated in heat-stressed pollen. Interestingly, we observed downregulation of genes homologous to *Arabinosyl transferase RRA3* (*BnaA06g13470D, BnaA06g13630D*) in heat-stressed pollen. In Arabidopsis, *RRA3* is involved in glycosylation of cell wall proteins which is suggested to be a crucial step in cell wall assembly for root hairs. This gene is also suggested (Velasquez et al., 2011) to be possibly involved in a similar modification of cell wall proteins involved in pollen tube growth but has not been experimentally validated. We also identified one *expansin* gene (*BnaCnng40260D*) downregulated by heat stress in pollen and pistil, which might play a role in cell wall loosening and assisting in penetration of stigma by the pollen tube (Valdivia et al., 2009). We conclude that downregulation of genes associated with cell wall organization and cellular transport adversely impact the pollen fitness and integrity, leading to reduced reproductive success and seed set.

### High Temperature-Induced Oxidative Stress Interferes With Reproductive Functions

In heat-stressed pollen and pistil, we identified genes associated with GO categories “response to hydrogen peroxide,” “response to reactive oxygen species” (Supplementary Tables S3e, S4e). Majority of the ROS responsive DEGs in pollen were also differentially regulated in pistil except for *BnaC03g73200D, BnaA09g36810D, BnaAnng06520D, BnaA02g06590D, BnaA06g18310D*, and *BnaC03g55730D*. Interestingly, homologs of NADPH oxidases (Respiratory burst oxidase homolog H and J), *BnaA02g06590D* (*RbohJ*), *BnaA06g18310D* (*RbohJ*), and *BnaC03g55730D* (*RbohH*) were downregulated only in heat-stressed pollen. In Arabidopsis, defective pollen tube growth which interrupts adequate fertilization was reported in the *rbohHrbohJ* double mutant, suggesting the role of these two genes in pollen germination (Boisson-Dernier et al., 2013). Furthermore, *RbohH* and *RbohJ* are involved in enzymatic production of ROS, which is crucial for pollen germination (Kaya et al., 2014). Thus, the downregulation of these genes negatively impacts the pollen function. Furthermore, the interaction network of RBOHs, ROS and Ca^2+^ concentrations is also possibly involved in pollen tube growth, and the negative regulation of this interaction by heat stress can cause interference with reproduction. Additionally, Ca^2+^ signaling is reported to play a crucial role in pollen tube growth and development (Konrad et al., 2018). The identified DEGs in heat-stressed pollen and pistil with a possible role in regulating Ca^2+^ gradient and signaling were homologous to Calcium-dependent lipid-binding (CaLB) family proteins, Calmodulin- binding family proteins, CML47, Calmodulin 1, Calcium-binding EF-hand family protein, Calcium-dependent protein kinase (CDPK), sodium/calcium exchangers, Calcium-transporting ATPase, alpha-mannosidase 3, Cyclic nucleotide-gated channel-16 (CNGC16) and others (Supplementary Tables S3f, S4f). We observed downregulation (log_2_foldchange 1.8) of a probable CNGC16 *B. napus* gene (*BnaCnng14420D*) in heat-stressed pollen. CNGC16 is crucial pollen expressing gene playing a role in heat stress-responsive Ca^2+^ signaling and downstream transcriptional heat stress response (Tunc-Ozdemir et al., 2013). The transcriptome analysis of the Arabidopsis mutant *cngc16* pollen highlighted the defect in triggering or maintaining heat stress-responsive transcriptome, indicating the importance of CNGC16 in pollen heat stress response (Ishka et al., 2018).

In heat-stressed pollen and pistil, aldehyde oxidase genes homologous to *glyoxal oxidase 1* (*Glox1*) were downregulated. In Arabidopsis, *Glox1* possibly play a vital role in the tapetum and pollen development (Krishnamurthy, 2015). It can be suggested that GLOX1 might negatively regulate pollen fitness and also play a damaging role in heat stress exposed pistil as well. Additionally, in heat-stressed pollen and pistil homologs of *galactinol synthase 1* (*GloS1*) and raffinose synthase family proteins were upregulated. These genes are involved in the raffinose family of oligosaccharides (RFOs) synthesis and have a possible role in oxidative stress response (Sengupta et al., 2015). *B. napus* genes homologous to ROS scavenging genes APX1 (Ascorbate Peroxidase 1; *BnaA06g04380D, BnaC05g05550D*) and APX2 (Ascorbate Peroxidase 2; *BnaA01g32160D, BnaC01g39080D*) were upregulated in pollen and pistil. The role of GLOS1 and raffinose synthase family proteins, APXs in heat stress response in reproductive tissues is not clear.

### Transcriptional Cascades Involved in Heat Stress Response in Pollen and Pistil

**FIGURE 9:**
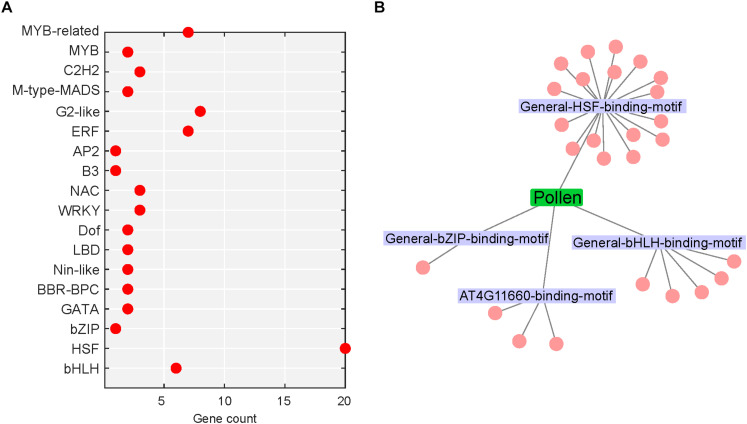
Heat stress-regulated transcriptional cascade in pollen. **(A)** TFs belonging to different TF gene families differentially regulated in heat-stressed pollen; red dot represents upregulated TFs. **(B)** Network of enriched TFs and the associated motifs present in the promoters of heat-responsive genes in heat-stressed pollen; the motifs are labeled, and the cluster associated with a motif represents the TFs.

**FIGURE 10:**
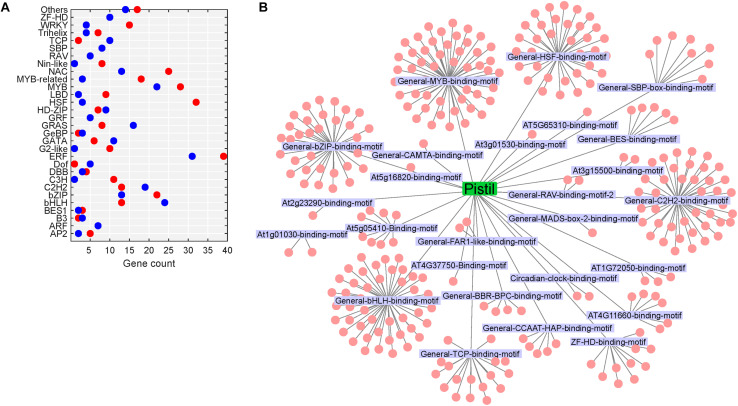
Heat stress-regulated transcriptional cascade in the pistil. **(A)** TFs belonging to different TF gene families differentially regulated in heat-stressed pistil; red dot represents upregulated TFs, and the blue dot represents downregulated genes. **(B)** Network of enriched TFs and the motifs present in the promoters of heat-responsive genes in heat-stressed pistil; the motifs are labeled, and the cluster associated with a motif represents the TFs.

Transcription factors (TFs) are fundamental to the regulation of gene expression. The transduction of stress signal to stress-responsive gene expression is mediated by TFs, which then interact with cis-acting elements located in the promoters of several target stress-responsive genes (Ohama et al., 2017). Around 4.9% (74/1,524; up: 74) and 7.9% (561/7,133; up: 305, down: 256) DEGs in heat-stressed pollen and pistil, respectively, were identified as encoding TFs (Figures 9A, 10A). Further, the SeqEnrich analysis, DNA sequence motifs or transcription factor binding sites (TFBS) significantly (*p* < 0.001) enriched in promoters of query genes are associated with TFs within the same query gene list capable of binding to that DNA sequence motif. Thus, we further identified the enriched transcriptional module, including TFs and their binding sites active in heat stress response in pollen and pistil (Figures 9B, 10B).

**FIGURE 11:**
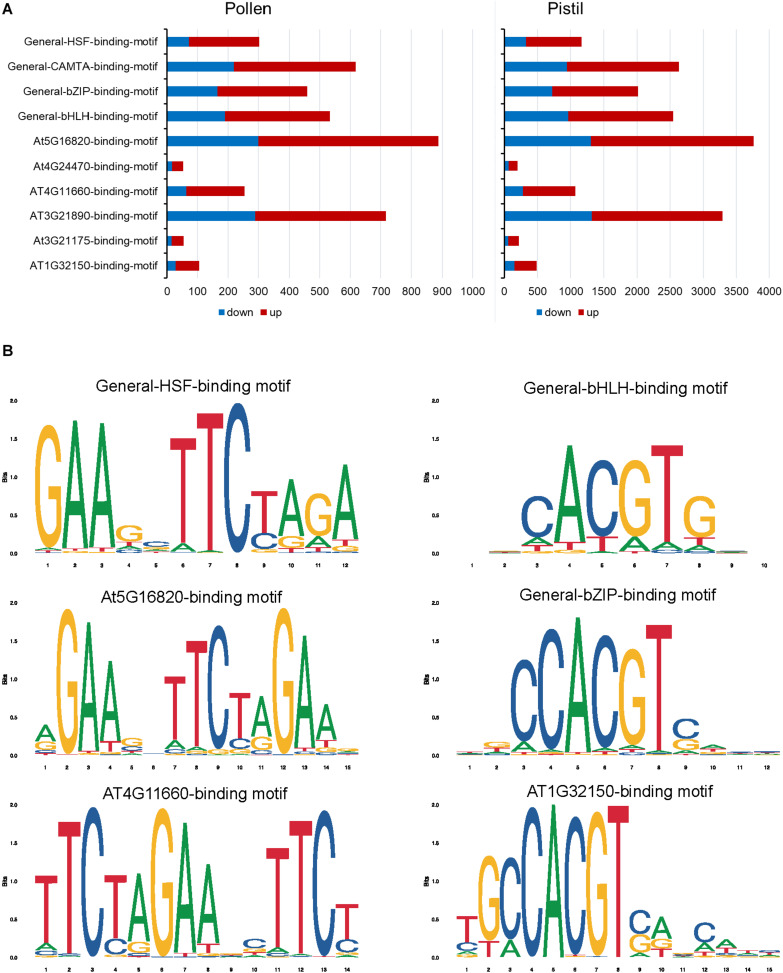
Heat stress-responsive transcription factor binding sites present in the 1 kb promoter region of the differentially regulated genes in heat-stressed pollen and pistil. **(A)** Top significantly enriched of DEGs in heat-stressed pollen and pistil. **(B)** Consensus sequence of HSF (General-HSF-binding-motif, At5g16820-binding-motif, At4G11660-binding-motif), bHLH and bZIP (General-bZIP-binding-motif, At1G32150-binding-motif) transcription factor binding sites.

In heat-stressed pollen and pistil, 13 and 47 significantly enriched TFBS were identified in the 1kb promoter region of the differentially expressed genes, respectively (Supplementary Tables S5, S6). The HSF binding motifs, bHLH binding motif, bZIP binding motif, GATA/TIFY binding motifs, MYB binding motifs and CAMTA binding motif were among the most significant TFBS predicted to be present in the target DEG’s promoters in heat-stressed pollen and pistil (Figure 11). The HSF binding sites (General-HSF-binding-motif, At5g16820-binding-motif, At4G11660-binding-motif) were present in 68 and 62% target genes in heat-stressed pollen and pistil, respectively. HSFs are well known to bind to the heat stress elements (HSEs: 5′-nGAAnnTTCnnGAAn-3′ or 5′-nTTCnnGAAnnTTCn-3′) present in the promoters of their target genes (Guo et al., 2016). In heat-stressed pollen and pistil, almost 35% of the target DEGs had the bHLH TFBS in their promoters. The bZIP TBFS (General-bZIP-binding-motif, At1G32150-binding-motif) were present in 32 and 31% DEGs in heat-stressed pollen and pistil. The consensus sequences of bZIP and bHLH TFBS had an ACGT core, and the bZIP and bHLH TFs are generally thought to recognize ACGT core containing ABRE sequences such as the G-box motif with flanking regions varying to some degree (Mehrotra et al., 2013; Ezer et al., 2017). The transcription factors bZIPs and bHLHs can act antagonistically, competing for binding to the same sites. Thus, understanding how bZIP and bHLH regulate their target genes is vital to explore pathways that can be engineered for imparting reproductive thermotolerance (Ezer et al., 2017).

In heat-stressed pollen, four significant TFBS were associated with 26 significantly enriched TFs. Among the enriched TFs, 19 genes belonged to the HSF family; five genes belonged to the bHLH family and one candidate to the bZIP family (Supplementary Table S7). *BnaC03g26940D*, *BnaA09g40360D*, *BnaC07g32600D*, *BnaA03g41550D*, and *BnaC03g52080D* with >11 log2 fold change in expression were among the top upregulated TFs. One transcription factor *BnaA04g09700D* showing association with the HSF-binding motif had no significant similarity with any Arabidopsis transcription factor. Interestingly no downregulation of any TF in heat-stressed pollen was observed.

In heat-stressed pistil 284 (up 144, down: 140) significantly enriched TFs were identified. The enriched temperature-responsive TFs in heat-stressed pistil belonged to diverse TF families such as bHLH, bZIP, HSF, DREB2A, AP2/ERF, MYB, NAC, WRKY, C2H2-zinc-finger, HD-zip (homeodomain-leucine zipper), FAR1-like (far-red impaired response-like) and transcription factors involved in brassinosteroids signaling, among others (Supplementary Table S8). Majority of the genes belonged to the MYB, bHLH, bZIP and HSF gene family. MYB (50 TFs) and MYB-related (21 TFs) TF members were significantly overrepresented in the heat-stressed pistil. Genes encoding MYB and MYB-related TFs were reported to be strongly induced by heat stress in maize pollen (Begcy et al., 2019). While the functions of MYBs in heat stress response in reproductive tissues are not clear, it has been reported that over-expression of wheat MYB transcription factor gene *TaMYB80* in Arabidopsis led to enhanced heat and drought stress tolerance (Zhao et al., 2017).

As discussed earlier, our results show that the *B. napus* HSFA2 (*BnaC03g26940D, BnaA03g22890D*), HSFA7 (HSF7a: *BnaA03g41550D, BnaC07g32600D, BnaA03g41540D*, HSF7b: *BnaA09g40360D*) and HSFB2a (*BnaC03g52080D*) were the key HSFs upregulated in both pollen and pistil with >100-fold change in expression levels. HSFA2 is reported to regulate thermotolerance during male reproductive development in tomato (Giorno et al., 2010; Fragkostefanakis et al., 2016). The heat stress exposure during tomato pollen development leads to enhanced *HsfA7* expression only in the tetrad stage, while enhanced expression of *HsfA2* was noticeable in all developmental stages (Keller et al., 2018). Class B HSFs acts as repressors of the expression of other HSFs. HSFB2a has been suggested to play a role in female gametophyte development (Wunderlich et al., 2014). In the present study, heat-stressed pollen, and pistil, bHLH TF family member, PIF6 (*BnaA09g39670D, BnaC08g32020D*) homologs showed >64-folds upregulation. In heat-stressed pollen, only *BnaC08g32020D* was upregulated, whereas in pistil both copies of PIF6 were upregulated. While PIF6 has been reported to act as a positive regulator of photomorphogenesis in plants and unlike other PIFs and it also plays a role in controlling seed dormancy (Pham et al., 2018), its role in thermo-sensing is not clear and remains an interesting area for future investigations.

In our data, heat-stressed pistil, down-regulated TFs, homologs of MYB75 (*BnaCnng28030D*), NYF subunit A3 (*BnaC04g27090D*), C2H2 type Zn finger protein (*BnaCnng02770D*), bHLH51 (*BnaA04g22490D*), and HSFC1 (*BnaC07g07130D*) were the top candidates with >40-fold decrease (Supplementary Table S8). In *Arabidopsis*, downregulation of gene expression of MYB75 (Georgii et al., 2017) and HSFC1 (Swindell et al., 2007) in response to heat stress has been reported. However, the causative relationship between the downregulation of these genes with heat stress response has not been established. Further, down-regulation of TFs in pistil as seen in our analysis, suggests that the heat stress-induced repression of transcription might be an adaptive mechanism that is markedly different between pollen and pistil. The significant transcriptional changes in gene expression in pollen and pistil in response to heat stress are largely due to the differential regulation of TF expression. Our study highlights the need for further investigations to understand the role of the network involving different HSFs along with other significant TFs in regulating the plant’s reproductive heat stress response in an organ/stage-specific manner.

## Conclusion

**FIGURE 12:**
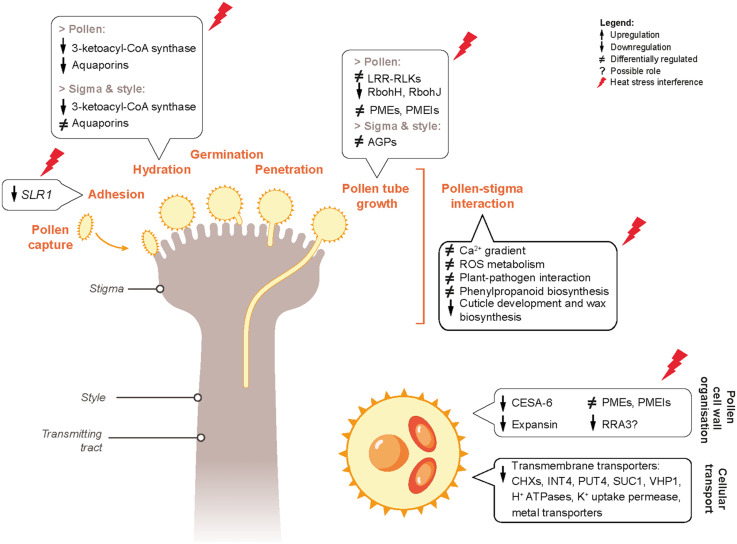
Proposed predictive gene regulatory model incorporating the genes and pathways differentially regulated by heat stress in mature pollen and pistil, resulting in negative regulation of pollen and pistil reproductive fitness and pollen-pistil interactions.

In this study, the effects of short-term heat stress events during flowering on the success of reproduction in *B. napus* have been described. We have shown that exposure to heat stress impairs the function of both pollen and pistil, leading to loss of fertility and seed set. The genes with possible involvement in negative regulation of pollen fitness and pollen-pistil interactions have also been identified. Based on the heat-responsive DEGs in pollen and pistil associated with heat-sensing, signaling cascades, cellular and metabolic processes; we propose a molecular mechanism through which heat stress possibly alters pollen-pistil interactions in *B. napus* (Figure 12). Our data reveal that detailed exploration of the molecular basis of these pathways is warranted. Dynamic transcriptomic analysis of non-stressed and heat-stressed pollen and pistil provided the basis of highlighting the distinctive mechanisms involved in heat stress response in these reproductive tissues. Further investigations on the DEGs identified in this study could be directed toward improving the performance and thermotolerance of *B. napus* varieties. Overall, data reported in our study provides a framework for further studies to explore key components involved in early heat sensing and response mechanisms during the reproductive phase of the Brassica crop.

## Data Availability Statement

All datasets generated for this study are included in the article/Supplementary Material. The RNA-Seq data generated for the analysis are deposited at the NCBI Sequence Read Archive (BioProject ID: PRJNA666230, BioSample IDs: SAMN16286967 and SAMN16286968). The output files generated by Kallisto analysis, containing the information of abundance estimates (counts and TPM), and transcript length information length for all samples analyzed in this study are provided in Supplementary Tables S9a–d.

## Author Contributions

NL conducted the experiments, analyzed the sequencing data, and prepared a draft of the manuscript. PB and MS conceived the research, supervised, and extensively edited the article. All authors contributed to the article and approved the submitted version.

## Conflict of Interest

The authors declare that the research was conducted in the absence of any commercial or financial relationships that could be construed as a potential conflict of interest.

## Footnotes

1 http://www.genoscope.cns.fr/brassicanapus/data/

2 http://jaspar.genereg.net/
