# Supplementary material for: RNA-Seq Highlights Molecular Events Associated With Impaired Pollen-Pistil Interactions Following Short-Term Heat Stress in Brassica napus
Source: Front Plant Sci. 2021 Jan 7;11:622748. doi: 10.3389/fpls.2020.622748 (PMC7872974; doi:10.3389/fpls.2020.622748)
Supplement: Supplementary file 1 [file Data_Sheet_1.PDF]

(a)

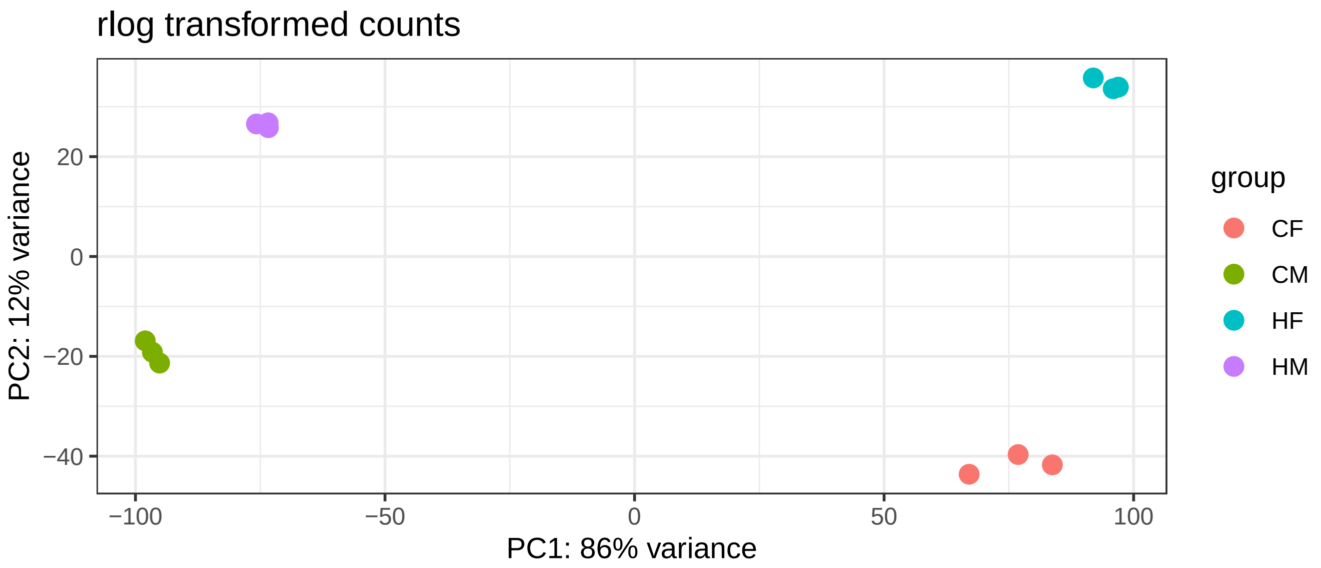

(b)

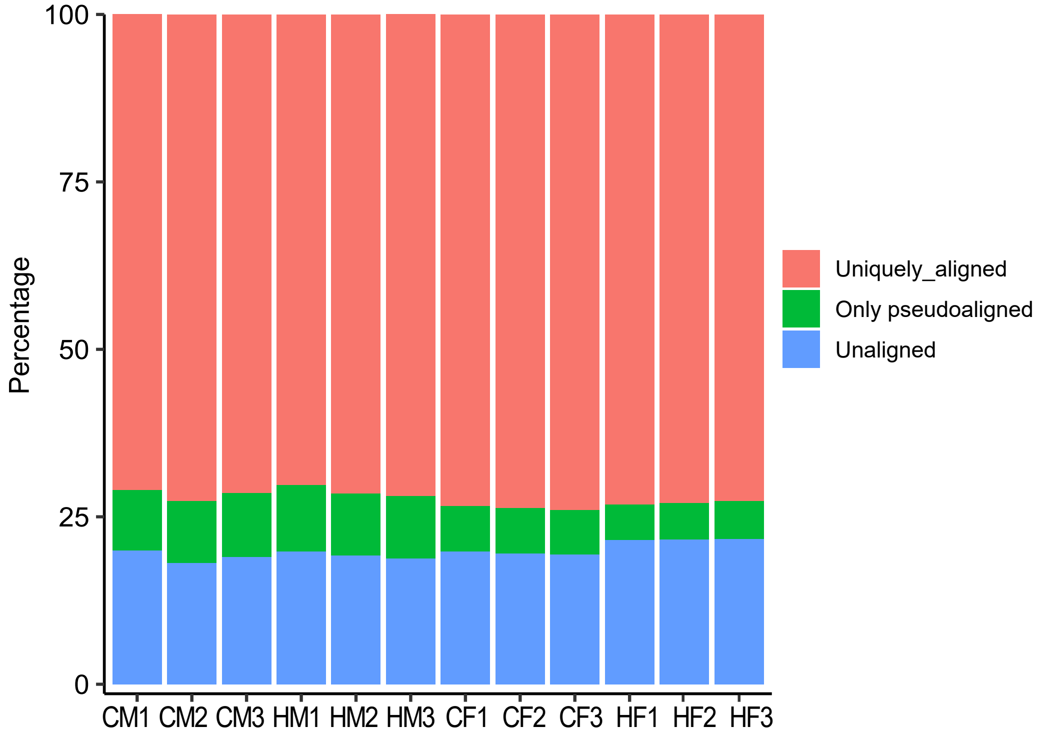

FIGURE S1 (a) PCA plot to illustrate the relatedness of replicates for each sample; CF: non-stressed pistil, HF: heat-stressed pistil, CM: non-stressed pollen, HM: heat-stressed pollen; **(b)** Alignment of clean reads to the reference transcript sequences in CM (non-stressed pollen), HM (heat-stressed pollen), CF (non-stressed pistil), and HF (heat-stressed pistil) libraries; (1=biological replicate 1, 2=biological replicate 2, 3=biological replicate 3).

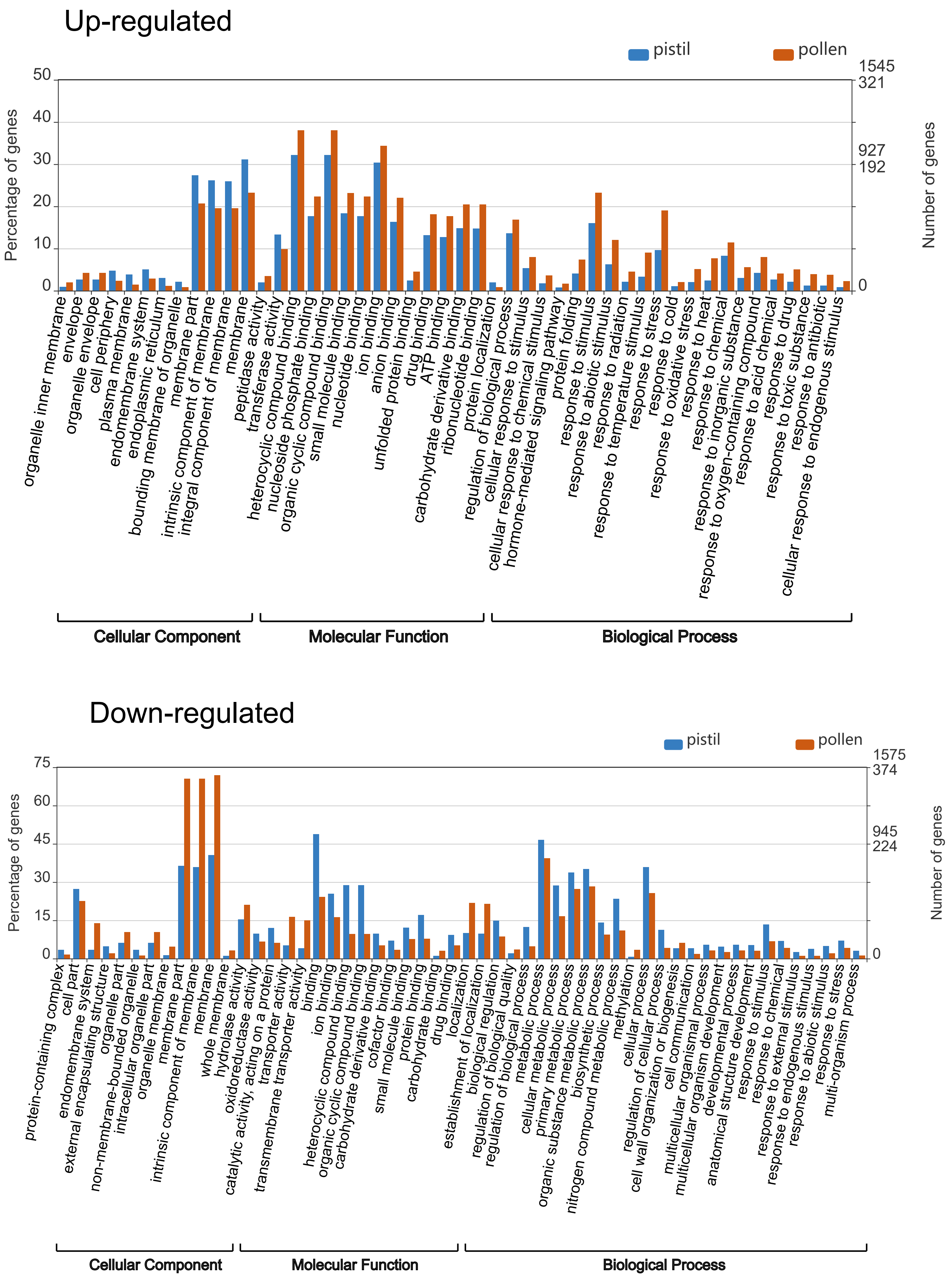

FIGURE S2 Details of GO annotation of up- and down- regulated DEGs in heat-stressed pollen and pistil.

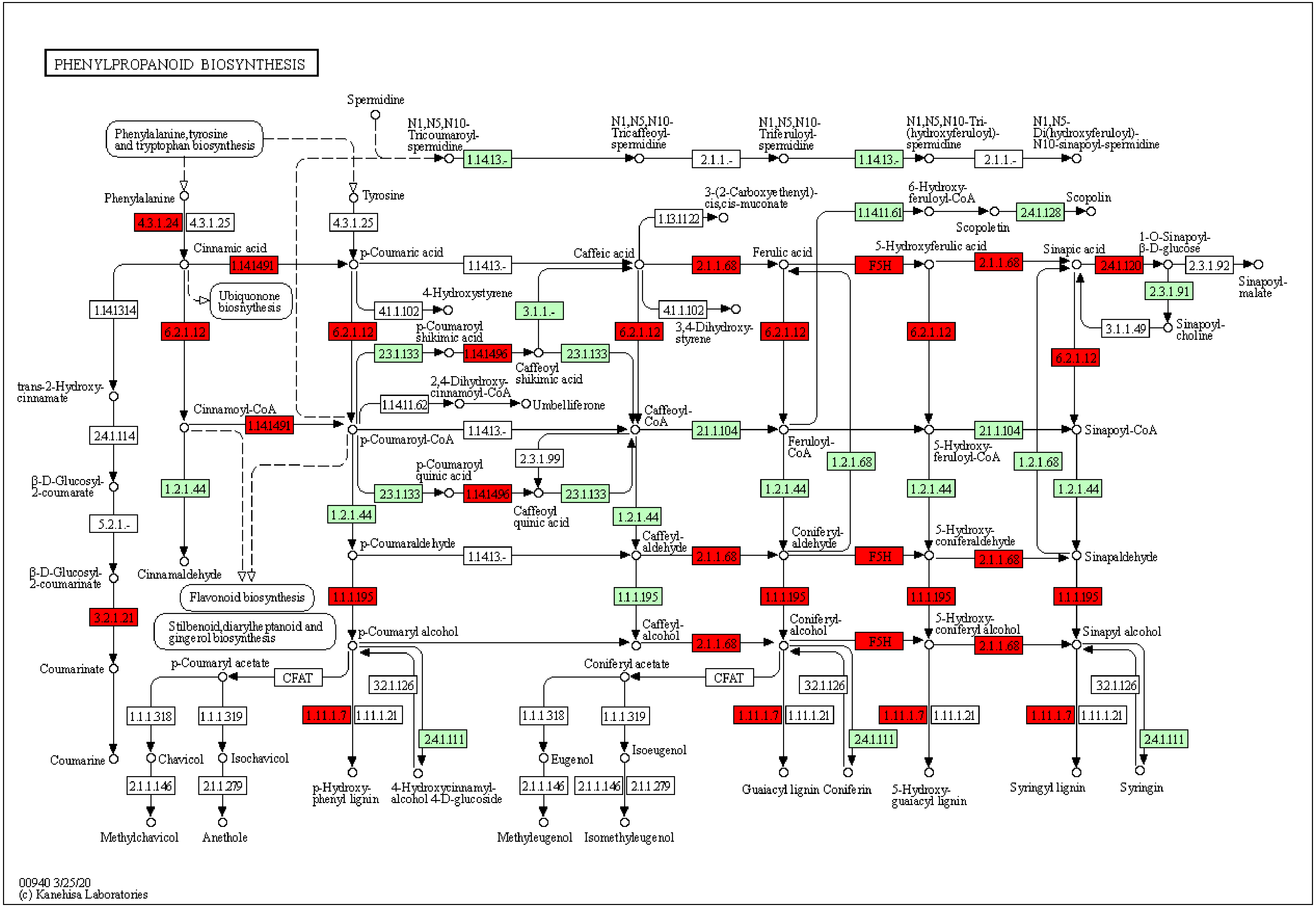

FIGURE S3 Differential regulation of "phenylpropanoid biosynthesis" pathway in heat-stressed pistil. The red boxes indicate the involvement of a DEG.
